# The TbD1 Locus Mediates a Hypoxia-Induced Copper Response in *Mycobacterium bovis*

**DOI:** 10.3389/fmicb.2022.817952

**Published:** 2022-04-14

**Authors:** Ruoyao Ma, Damien Farrell, Gabriel Gonzalez, John A. Browne, Chie Nakajima, Yasuhiko Suzuki, Stephen V. Gordon

**Affiliations:** ^1^UCD School of Veterinary Medicine, University College Dublin, Dublin, Ireland; ^2^Hokkaido University International Institute for Zoonosis Control, Sapporo, Japan; ^3^UCD School of Agriculture and Food Science, University College Dublin, Dublin, Ireland; ^4^Division of Bioresources, Research Center for Zoonosis Control, Hokkaido University, Sapporo, Japan; ^5^UCD Conway Institute, University College Dublin, Dublin, Ireland

**Keywords:** tuberculosis, transcriptomic (RNA-seq), *Mycobacterium bovis*, phenotype, hypoxia, TbD1

## Abstract

The *Mycobacterium tuberculosis* complex (MTBC) contains the causative agents of tuberculosis (TB) in mammals. The archetypal members of the MTBC, *Mycobacterium tuberculosis* and *Mycobacterium bovis*, cause human tuberculosis and bovine tuberculosis, respectively. Although *M. tuberculosis* and *M. bovis* share over 99.9% genome identity, they show distinct host adaptation for humans and animals; hence, while the molecular basis of host adaptation is encoded in their genomes, the mechanistic basis of host tropism is still unclear. Exploration of the *in vitro* phenotypic consequences of known genetic difference between *M. bovis* and *M. tuberculosis* offers one route to explore genotype–phenotype links that may play a role in host adaptation. The TbD1 (“*Mycobacterium tuberculosis* deletion 1 region”) locus encompasses the *mmpS6* and *mmpL6* genes. TbD1 is absent in *M. tuberculosis* “modern” lineages (Lineages 2, 3, and 4) but present in “ancestral” *M. tuberculosis* (Lineages 1 and 7), *Mycobacterium africanum* lineages (Lineages 5 and 6), newly identified *M. tuberculosis* lineages (Lineages 8 and 9), and animal adapted strains, such as *M. bovis*. The function of TbD1 has previously been investigated in *M. tuberculosis*, where conflicting data has emerged on the role of TbD1 in sensitivity to oxidative stress, while the underlying mechanistic basis of such a phenotype is unclear. In this study, we aimed to shed further light on the role of the TbD1 locus by exploring its function in *M. bovis*. Toward this, we constructed an *M. bovis* TbD1 knockout (ΔTbD1) strain and conducted comparative transcriptomics to define global gene expression profiles of *M. bovis* wild-type (WT) and the ΔTbD1 strains under *in vitro* culture conditions (rolling and standing cultures). This analysis revealed differential induction of a hypoxia-driven copper response in WT and ΔTbD1 strains. *In vitro* phenotypic assays demonstrated that the deletion of TbD1 sensitized *M. bovis* to H_2_O_2_ and hypoxia-specific copper toxicity. Our study provides new information on the function of the TbD1 locus in *M. bovis* and its role in stress responses in the MTBC.

## Introduction

Tuberculosis (TB) caused by *Mycobacterium tuberculosis* is one of the leading causes of death worldwide due to a single infectious agent. TB killed an estimated 1.5 million people in 2020 alone, with an increase in TB deaths for the first time in over a decade due to the disruption to TB control programs caused by the COVID-19 pandemic [World Health Organization [WHO], 2021]. Bovine tuberculosis (bTB) is mainly caused by *Mycobacterium bovis*, however, *M. bovis* can also infect and sustain in many other domesticated and wild mammalian hosts, such as goats, badgers, deer, and feral water buffalo (Gormley and Corner, 2018). *M. bovis* is also a zoonotic risk in terms of transmission of infection from animals to humans, albeit *M. bovis* has a limited transmission capacity between immune competent humans compared to *M. tuberculosis* (Francis, 1950; Magnus, 1966). On the other hand, modern strains of *M. tuberculosis* appear attenuated in cattle compared to *M. bovis* and seem unable to sustain in cattle populations, although there are reported cases of potential reverse zoonotic transmission of *M. tuberculosis* to animals (Whelan et al., 2010; Ameni et al., 2011). The archetypal MTBC strains *M. tuberculosis* H37Rv and *M. bovis* AF2122/97 show more than 99.9% genome sequence identity, in spite of the distinct host preference displayed by the bacilli (Garnier et al., 2003). Elucidating the molecular basis of MTBC host preference will help to reveal not only the evolutionary steps that led to the emergence of these pathogens but may also reveal new ways to control TB in animals and humans.

The *M. tuberculosis* specific deletion one region (TbD1) is a 2153 bp DNA region that was originally identified through comparative genomics between the *M. tuberculosis* H37Rv genome with other MTBC members (Brosch et al., 2002). TbD1 was used as a marker of so-called “ancient” and “modern” lineages, whereby MTBC strains having TbD1 intact were deemed as having the ancestral configuration of this locus. The TbD1 locus encodes two proteins: the putative membrane protein MmpS6 and a transmembrane transport protein MmpL6. MmpL are “mycobacterial membrane protein large” proteins that belong to the resistance-nodulation-cell division (RND) efflux pump families and perform functions as diverse as transport of lipids, uptake of nutrients, iron acquisition, or extrusion of toxic compounds (Wells et al., 2013; Delmar et al., 2015; Melly and Purdy, 2019). The genome of *M. tuberculosis* H37Rv encodes 13 MmpL proteins (Cole et al., 1998) which have been classified into hydrophobe/amphiphile efflux (HAE) subfamilies of RND based on phylogenetic comparisons; hence, MmpL1, 2, 4, 5, 6, 8, 9, 10, and 12 belong to the HAE2 family while MmpL3, 7, and 11 belong to HAE3 family (Sandhu and Akhter, 2015). In *Escherichia coli* the HAE-1 RND transporter AcrB is only active in conjunction with membrane fusion proteins (MFPs) and outer membrane factors (OMFs) that form an elongated tripartite complex, AcrAB(ZZ)-TolC (Kobylka et al., 2020). In mycobacteria, the MmpS proteins are speculated to function in a similar way to MFPs, but their precise role remains to be defined (Deshayes et al., 2010; Wells et al., 2013).

Five RND transporters with known structures are AcrB, CusA, MexB, ZneA, and MtrD. These proteins have 12-helix transmembrane domains with a periplasmic N-terminal porter (PN) subdomain located between TM1 and TM2, and a periplasmic C-terminal porter (PC) subdomain inserted between TM7 and TM8 (Murakami et al., 2002; Sennhauser et al., 2009; Long et al., 2010; Su et al., 2012; Nakashima et al., 2013; Pak et al., 2013). In *M. tuberculosis* H37Rv, MmpL1, 2, 3, 4, 5, 7, 8, 9, 10, 11, and 12 have 11–12 TMs and one or two large periplasmic loops that make up the porter domain. In all human-adapted modern *M. tuberculosis* lineages, including major epidemic strains such as those of Lineage 2 (Beijing) and Lineage 4 (Euro-American Haarlem) (Hershberg et al., 2008; Wirth et al., 2008), TbD1 is deleted with the *mmpL6* remnant encoding a protein with only five TMs that lacks the periplasmic porter domain. This suggests that MmpL6 is not functional in TbD1-deficient mycobacterial strains. However, *M. tuberculosis* strains from lineages with an intact TbD1 locus, as well as animal-adapted lineages including *M. bovis*, encode MmpL6 harboring 12 TMs, and two porter subdomains: PN and PC (Sandhu and Akhter, 2015).

It has been suggested that the loss of TbD1 gave a selective advantage to modern *M. tuberculosis* strains (Reiling et al., 2013; Bottai et al., 2020). This is supported by the fact that modern strains trigger an attenuated inflammatory host response and have an enhanced ability to grow in human macrophages, as well as an association with increased bacillary loads in the lungs of infected mice compared to ancestral strains that contain an intact TbD1 locus (Portevin et al., 2011; Reiling et al., 2013; Bottai et al., 2020). Also, distinct geographic distributions for ancestral and modern strains have been found by several studies, e.g., ancestral strains in Lineage 1 are found mainly in countries bordering the Indian Ocean, while modern strains have a wider global distribution (Merker et al., 2015).

There have been two main studies that have explored the function of the TbD1 locus. Arumugam et al. (2019) showed that oxidative stress can trigger the expression of *mmpS6* and *mmpL6* genes, and the presence of the intact TbD1 locus afforded a higher tolerance to oxidative stress for *M. tuberculosis*. On the other hand, Bottai et al. (2020) presented evidence that loss of TbD1 conferred protection against oxidative stress.

We sought to explore the function of TbD1 in *M. bovis* as a route to determine its function in the archetypal animal-adapted tubercule bacillus. We constructed *M. bovis* TbD1 knockout and complemented mutants and explored transcriptomic alterations between the TbD1 mutant and wild-type (WT) under different *in vitro* culture conditions. These analyses revealed differential expression of genes involved in hypoxia-driven copper stress response between the *M. bovis* TbD1 mutant and WT. Subsequent *in vitro* experiments revealed a potential link between the TbD1 locus in *M. bovis* with a hypoxia-specific response to copper and oxidative stress. This work therefore reveals new insight into the functional significance of genetic variations between members of the MTBC, linking variation in the TbD1 locus to pathogen responses to *in vivo* relevant stresses.

## Materials and Methods

### Bacterial Strains and Culture Conditions

*Escherichia coli* strains that were used for plasmid propagation in MultiSite Gateway cloning (Life Technologies/Invitrogen/Thermo Fisher Scientific, Loughborough, United Kingdom) procedures were grown in LB medium or LB agar plates supplemented with selected antibiotics. Ampicillin (50 μg/ml), zeocin (25 μg/ml), and hygromycin (50 μg/ml) were added as required. BCG Denmark was grown in liquid Middlebrook 7H9 medium (Becton Dickinson, New Jersey, NJ, United States) supplemented with 0.05% Tween 80, 0.2% glycerol, 0.5% bovine serum albumin, 0.2% glucose, and 0.085% NaCl or 7H11 agar plates (Becton Dickinson, New Jersey, United States) supplemented with 0.2% glycerol, 0.5% BSA, 0.2% glucose, and 0.085% NaCl. *M. bovis* AF2122/97 was grown in the 7H9 and 7H11 media as described above, with 40 mM sodium pyruvate (Sigma-Aldrich, Ireland). When required, kanamycin, hygromycin, or zeocin were added to growth media to a final concentration of 50, 50, or 25 μg/ml, respectively. Standing cultures for RNA extraction were grown in 30 ml of 7H9 in 50 ml tubes (Sarstedt) with the caps tightly screwed, without shaking at 37°C. Rolling cultures for RNA extraction were grown in 30 ml of 7H9 in 850 cm^2^ roller bottles (Cellmaster), rolling at 2–3 rpm at 37°C. Sauton’s medium was prepared using 4 g L-asparagine, 2 g citric acid, 0.5 g KH_2_PO_4_, 0.5 g MgSO_4_, 0.05 g ferric ammonium citrate, 0.1 ml of 0.01% ZnSO_4_, 60 ml glycerol, 2.5 ml of 20% Tween 80 in 900 ml deionized water and adjust pH to 7.0 with 1 M NaOH, adding 40 mM sodium pyruvate for *M. bovis* AF2122/97. The strains used in this study are listed in Supplementary Table 1.

### Mutant Generation and Complementation

*Mycobacterium bovis* AF2122/97 and BCG Demark ΔTbD1 mutants were constructed using the Che9c recombineering system (van Kessel and Hatfull, 2007). Briefly, pNitET-SacB-kan plasmids were transformed into *M. bovis* strains by electroporation to create recombinogenic *M. bovis* strains. During the allelic exchange process, the hygromycin cassette from the allelic exchange substrate (AES) that was constructed *via* MultiSite Gateway (Life Technologies/Invitrogen/Thermo Fisher Scientific, Loughborough, United Kingdom) replaced TbD1. Knockouts were selected on 7H11 plates containing hygromycin. PCR was used for initial mutant screening for verification of the TbD1 deletion and presence of the hygromycin cassette. The left and right junction arms of the hygromycin cassette in ΔTbD1 were amplified by PCR and Sanger sequenced to rule out rearrangements in neighbor genes. The spontaneous loss of pNitET-SacB-kan plasmid was also verified by PCR. The complemented *M. bovis* strains were constructed using integrative plasmid pDE43-MCZ-TbD1 that were also produced using the Gateway system; these plasmids integrate into conserved *attB* site on the mycobacterial genome (Saviola and Bishai, 2004), allowing stable expression of the TbD1 locus under the native promoter. The colonies were checked by PCR to verify the rescue of the TbD1. The plasmids and primers used in this study are listed in Supplementary Table 1.

### RNA Extraction, RNA-seq, and RT-qPCR

For RNA-seq, *M. bovis* AF2122/97 WT and ΔTbD1 strains were grown in triplicate in 7H9 medium in rolling or standing conditions to OD_600_ = 0.4–0.6. RNA samples were extracted as previously described (Malone et al., 2018). RNA concentration was determined using the NanoDrop One spectrometer (Thermo Scientific, Massachusetts, MA, United States) and RNA integrity number (RIN) was determined by RNA 6000 Nano kit (Agilent Technologies, Cork, Ireland) on the Bioanalyser 2100 (Agilent Technologies, Cork, Ireland). All RNA samples were shown to have RIN values over 8.5 and were sent for RNA-seq. Following rRNA removal using the Ribo-Zero rRNA kit (Illumina, California, CA, United States), strand-specific RNA libraries were prepared with an insert size of 250–300 bp using the NEBNext Ultra II Directional RNA Library Prep Kit (New England Biolabs, United Kingdom) and sequencing was performed by the commercial supplier (Novogene, Cambridge, United Kingdom) on an Illumina NovaSeq 6000 machine. Single-end, strand-specific 150 bp reads data were generated for *M. bovis* AF2122/97 WT and ΔTbD1 strains grown in standing condition and paired-end, strand-specific 150 bp reads data were generated for *M. bovis* AF2122/97 WT and ΔTbD1 strains grown in rolling condition. For RT-qPCR, RNA samples were extracted, and 1 μg of RNA was reverse transcribed to single-stranded cDNA using the High-Capacity cDNA Reverse Transcription Kit (Applied Biosystems/Thermo Fisher Scientific, Warrington, United Kingdom) according to the manufacturer’s guidelines, minus RT controls and no-template controls were included. The cDNA was diluted to 2 ng/μl RNA equivalents. For qPCR (20.0 μl final volume), the master mix was prepared using 1.2 μl of forward/reverse primer (300 nM final), 10 μl SYBR Green Mix (Applied Biosystems/Thermo Fisher Scientific, Warrington, United Kingdom), 2.6 μl nuclease-free H_2_O, and 5 μl cDNA template. Duplicate qPCR was performed for each sample on the 7500 Fast Real-Time PCR System (Applied Biosystems/Thermo Fisher Scientific, Warrington, United Kingdom) with an initial denaturation at 95°C for 10 min, with 40 cycles of denaturation (95°C for 15 s), and a combined annealing/extension (60°C for 1 min), followed by a final melt curve, to confirm primer specificity. The qPCR data was analyzed using the qBASE + qbasePLUS software package.

### Differential Gene Expression Analysis of *Mycobacterium bovis* AF2122/97

Raw reads were processed firstly by FastQC tool (Babraham Bioinformatics, 2019a – FastQC A Quality Control tool for High Throughput Sequence Data) to check the quality and then Trim Galore (Babraham Bioinformatics, 2019b – Trim Galore!) was used to trim off reads with low-quality base calls and adapters. Bwa-mem (Li, 2013) were used to do the alignment between the raw reads with *M. bovis* AF2122/97 reference genome. FeatureCounts (Liao et al., 2014) was used for reads counting for each gene. The R package DESeq2 (Love et al., 2014) was used for differentially expressed gene (DEG) analysis with fold-change and adjusted *p*-value threshold. The analyses of protein–protein interaction and functional enrichment were performed by the STRING and were created by Cytoscape (Shannon et al., 2003; Szklarczyk et al., 2019).

### Drop Assays and Copper Challenge

For drop assays, BCG Denmark WT and TbD1 mutants were scaled up to OD_600_ = 0.8–1.0 as described above. Cultures were then pelleted by centrifugation and washed twice with Sauton’s media and resuspended in Sauton’s media to OD_600_ = 0.1. Ten-fold serial dilutions were conducted with each culture on a 96-well plate. Six-microliters drops were spotted onto 7H11 agar plates with increasing concentrations (25, 100, and 150 μM) of CuSO_4_ in replicates and incubated at 37°C for 14–16 days. For copper challenge in liquid media, cultures were collected, washed, and resuspended as described above and 150 μM CuSO_4_ was added. The OD_600_ were then read constantly over 14 days. To check the cell viability after copper stress was imposed on *M. bovis* AF2122/97, the strains were grown in 7H9 with 10 mM sodium pyruvate to OD_600_ = ∼0.8 and cultures were then pelleted by centrifugation, washed twice with Sauton’s media and resuspended in Sauton’s media to OD_600_ = 0.1. Cultures then were maintained in the absence (control) or presence of 200 μM CuSO_4_ in standing conditions for 10 days. Cultures were plated out at day 10 on 7H11 plates. CFU were determined after 2–3 weeks incubation. The viability was expressed as a percentage of survival, calculated as the ratio between the CFU recovered from cultures exposed to 200 μM CuSO_4_ over those obtained from unexposed cultures.

### H_2_O_2_ Challenge and CFU Determination

*Mycobacterium bovis* AF2122/97 WT and TbD1 mutants were cultured in 7H9 medium to mid-log phase and diluted to OD_600_ = 0.1. Ten millimolars and 40 mM of H_2_O_2_ were added and incubated at 37°C for 1 h and cultures were plated on 7H11 plates. After 2–4 weeks incubation at 37°C, CFU were enumerated. Susceptibility was expressed as a percentage of survival, calculated as the ratio between the CFU recovered from cultures exposed to stress over those obtained in unexposed cultures, multiplied by 100.

## Results

### Generation and Verification of *Mycobacterium bovis* TbD1 Mutants

**FIGURE 1:**
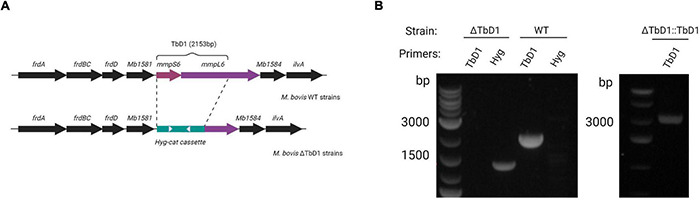
Construction of *M. bovis* ΔTbD1 mutant and complemented ΔTbD1:TbD1 strain. **(A)** Diagram showing the TbD1 and flanking genes. The TbD1 region was replaced by a 3.5 kB *hyg-cat* cassette (green bar) to generate *M. bovis* ΔTbD1 strains. White arrowheads indicate primer targets (Hyg F&R) to verify the Hyg cassette. **(B)** Agarose gel electrophoresis image after PCR with primers targeting TbD1 specific internal region and primers targeting *hyg-cat* cassette to verify the knockout of TbD1 and the presence of *hyg-cat* cassette on BCG Denmark ΔTbD1 strains and PCR targeting TbD1 locus to verify the complementation of ΔTbD1 on ΔTbD1:TbD1 strains.

TbD1 mutants were constructed in two *M. bovis* genetic backgrounds: *M. bovis* AF2122/97 and *M. bovis* BCG Denmark. ΔTbD1 mutants were constructed using the Che9c recombineering system, with TbD1 replaced by a hygromycin resistance cassette *via* allelic exchange (Figure 1A; van Kessel and Hatfull, 2007). PCR amplification of the TbD1 internal region verified the deletion of TbD1 and the replacement with the hygromycin cassette (Figure 1B). PCR amplification of the flanking regions and Sanger sequencing of the PCR fragments verified that there were no off-target mutations in the flanking regions. To perform trans-complementation of the TbD1 locus back into ΔTbD1 strains we constructed an integrative plasmid (pDE43-MCZ-TbD1) with expression of the TbD1 locus driven by native *mmpS6* promoter. The complemented mutant strains, termed ΔTbD1:TbD1, were verified by PCR to confirm the genomic integration of the TbD1 locus (Figure 1B).

### Comparative Transcriptome Analysis of *Mycobacterium bovis* AF2122/97 Under Standing and Rolling Culture Conditions

Standing culture conditions are known to induce a low-oxygen environment and hypoxic response in *M. bovis* BCG (Florczyk et al., 2001). Thus, we analyzed RNA-seq data from *M. bovis* AF2122/97 WT strains under rolling and standing conditions as a simple system in which to study what effects these different culture conditions would have on *M. bovis* global gene transcription, and how this might impact transcription at the TbD1 locus.

**FIGURE 2:**
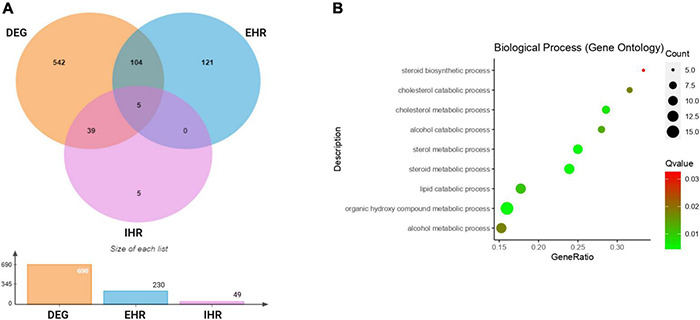
Comparative transcriptome analysis of *M. bovis* AF2122/97 WT under standing and rolling culture conditions. **(A)** Venn diagram showing the overlap of the genes shared among differentially expressed genes (DEG: orange)in *M. bovis* AF2122/97 standing cultures as compared to rolling cultures, enduring hypoxic response (EHR: blue) and initial hypoxic response (IHR: purple) gene lists. **(B)** Gene ontology (GO) enrichment analysis for differentially expressed genes in standing *M. bovis* AF2122/97. The gene ratio refers to the ratio of the number of genesin the GO entry following enrichment to the total number of genes in the GO entry. An increased gene ratio indicates greater enrichment. Lower *Q* value indicates higher significance.

By analysis of global transcription patterns from *M. bovis* AF2122/97 under standing and rolling culture conditions we obtained 179 DEGs after filtering with fold-change and *p*-value thresholds (|log_2_FC|> 2; *P*_*adj*_.<0.05). DEGs were then converted to *M. tuberculosis* H37Rv orthologs for comparison to *M. tuberculosis* annotations and the previous literature. Many of the DEGs that related to hypoxia/low oxygen responses (*acr*/*hspX*/*Rv2031c* and *acg*/*Rv2032*) were highly expressed in *M. bovis* AF2122/97 WT under standing as compared to rolling growth conditions. Among the DEGs, genes for some well-known transcription factors that mediate responses of *M. tuberculosis* to hypoxia, e.g., *dosR*, *Rv0081*, *Rv0324*, and *kmtR* were also found to be upregulated in *M. bovis* standing cultures (Galagan et al., 2013). Gene ontology (GO) functional analysis of the DEGs enriched GO terms included *Steroid biosynthetic process* (GO:0006694), *Cholesterol catabolic process* (GO:0006707), *Cholesterol metabolic process* (GO:0008203), *Alcohol catabolic process* (GO:0046164), *Sterol metabolic process* (GO:0016125), *Steroid metabolic process* (GO:0008202), *Lipid catabolic process* (GO:0016042), *Organic hydroxy compound* (GO:1901615), and *Alcohol metabolic process* (GO:0006066) (Figure 2B and Supplementary Tables 3, 6). The upregulation of genes under these GO terms showed an induction of lipid metabolism in *M. bovis* cultured under standing conditions.

Rustad et al. (2008) identified 230 enduring hypoxic response (EHR) genes that were stably induced by hypoxic stress and largely independent of the DosR regulon. To allow direct comparison with their data, we thus used the same log_2_FC threshold of |log_2_FC|> 1 and *P*_*adj*_.< 0.05 as they employed. These filters identified 109 genes from our DEG that overlapped with EHR genes (230 genes in total) and 44 overlapping genes with DosR regulated initial hypoxic response (IHR) genes (49 genes in total), showing a significant overlap between our standing conditions and hypoxic conditions (Figure 2A and Supplementary Table 2). This again indicated that the *M. bovis* AF2122/97 standing cultures had undergone hypoxic responses. Notably, the expression of *mmpL6* was approximately twofold higher in standing as compared to rolling conditions, indicating that expression of the TbD1 locus is induced in standing culture conditions.

### The Effects of TbD1 on Global Gene Expression of *Mycobacterium bovis* AF2122/97 Under Standing and Rolling Growth Conditions

**FIGURE 3:**
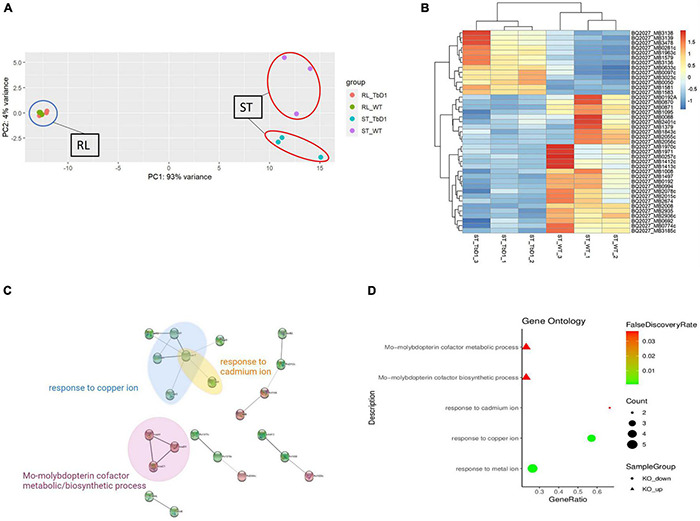
Comparative transcriptome analysis of *M. bovis* AF2122/97 WT and ΔTbD1 under standing growth condition and rolling growth condition. **(A)** Principal-component analysis (PCA) of the WT and ΔTbD1 *M. bovis* AF2122/97 transcriptomes from two different culture conditions: blue circle indicates the rolling condition and red circles indicate the standing condition. Each point represents a strain sample as indicated in the legend on the right. **(B)** Hierarchical clustering-heatmap of the 41 differentially expressed genes in *M. bovis* AF2122/97 ΔTbD1. Upregulated expression is presented in red and downregulated expression is presented in blue. Each row represents a gene, and each column represents a strain sample. **(C)** Protein–protein interaction network of the differentially expressed genes. Red nodes represent upregulated genes, while green nodes represent downregulated genes. The edges indicate the association between genes and edge width indicates the score of confidence of an interaction on the available evidence from STRING (the wider the edge, the higher scores of the interaction). **(D)** Gene ontology (GO) enrichment analysis for differentially expressed genes in *M. bovis* AF2122/97 ΔTbD1 grown in standing conditions. The GO enrichment terms from enrichment analysis of the upregulated (triangle) and downregulated (circle) genes are presented. The gene ratio refers to the ratio of the number of genes in the GO entry following enrichment to the total number of genes in the GO entry. An increased gene ratio indicates greater enrichment. Lower false discovery rate indicates higher significance.

The standing and rolling culture conditions provided us with an *in vitro* system to explore transcriptome differences between *M. bovis* AF2122/97 WT and ΔTbD1 strains. A principal-component analysis (PCA) plot (Figure 3A) of gene expression between *M. bovis* AF2122/97 WT and ΔTbD1 revealed separation of WT and ΔTbD1 standing culture groups, showing that the gene expression profiles of WT vs. ΔTbD1 *M. bovis* AF2122/97 were only significantly different when the bacilli were grown under standing conditions.

Given that *mmpL6* gene was upregulated in standing condition as stated above, indicated a “switch-on” of TbD1 locus and associated pathways by these conditions, we thus focused on standing cultures specifically to study the effects of the TbD1 locus on global gene expression in *M. bovis* AF2122/97. There were 41 DEGs meeting the thresholds applied (|log_2_FC|> 1 and *P*_*adj*_.< 0.05) in *M. bovis* AF2122/97 ΔTbD1 cultured with standing conditions (Figure 3B). The DEGs were converted to *M. tuberculosis* H37Rv orthologs to facilitate input into the STRING database (Szklarczyk et al., 2019) and hence allow analysis of the protein–protein interactions and GO enrichment in the DEG (Figure 3C and Supplementary Table 4). The enriched GO terms indicated that knockout of TbD1 led to differential expression of genes involved in *Response to copper ion* (GO:0046688), *Response to cadmium ion* (GO:0046686), *Response to metal ion* (GO:0010038), *Mo-molybdopterin cofactor biosynthetic process* (GO:0006777), and *Mo-molybdopterin cofactor metabolic process* (GO:0019720) (Figures 3C,D). Four (*mymT*, *lpqS*, *ctpV*, and *ctpG*) of the seven genes from the copper ion response GO term (GO:0046688) were significantly downregulated in the *M. bovis* AF2122/97 ΔTbD1 under standing growth conditions. Apart from the enriched genes mentioned above, other copper-responsive genes regulated by CsoR and RicR were significantly downregulated in the ΔTbD1 mutant under standing conditions (Supplementary Table 5).

### TbD1 Locus Is Involved in *Mycobacterium bovis* AF2122/97 Hypoxia-Specific Copper Response

**FIGURE 4:**
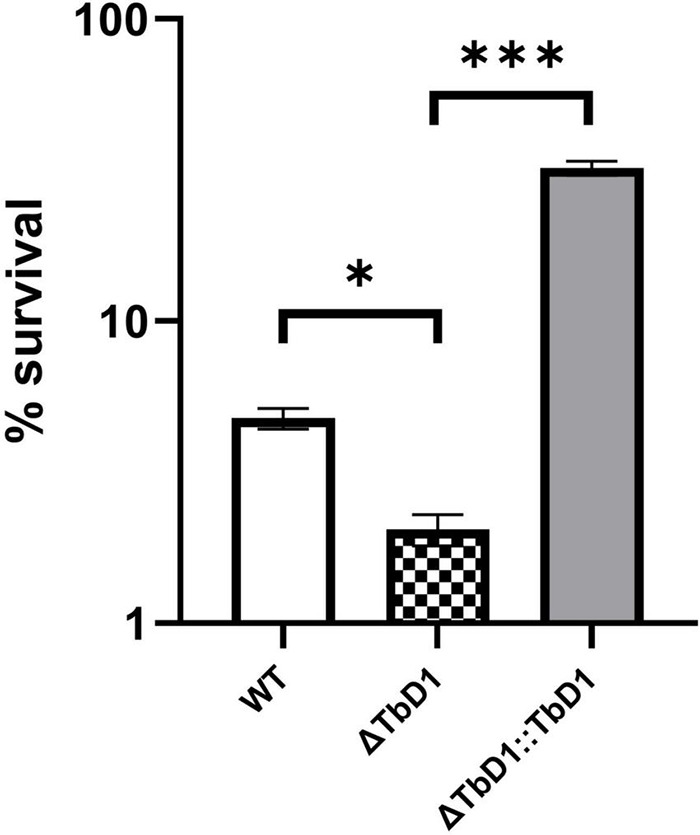
Copper stress experiments with *M. bovis* AF2122/97. Copper stress experiments were performed on *M. bovis* AF2122/97 WT, ΔTbD1, and ΔTbD1:TbD1 strains to assess the ability of TbD1 mutants to survive at high concentration of Copper ions. Two hundred micromolars of CuSO_4_ was added into standing cultures for 10 days without agitation and then cultures were plated on 7H11 plates with trace amounts of Copper. CFU were then counted after 2–3 weeks of incubation and survival percentages were determined relative to the non-CuSO_4_ control. The data are shown as means ± SD and are representative of three independent experiments. Statistical significance of differences in survival percentages calculated using one-way ANOVA followed by the Dunnett’s *post hoc* test for multiple-comparisons (*p < 0.05; ^***^p < 0.001).

Based on the transcriptomic data that revealed differential regulation of genes implicated in copper stress responses in the ΔTbD1 mutant, we then explored whether the TbD1 locus was implicated in the maintenance of copper homeostasis in *M. bovis* AF2122/97 when grown under our standing culture conditions. We thus grew *M. bovis* AF2122/97 WT, ΔTbD1, and ΔTbD1:TbD1 strains to OD_600_ = ∼0.8; cultures were washed and resuspended in Sauton’s media to OD_600_ = 0.1, then 200 μM of CuSO_4_ added to the “treated” group and incubated for another 10 days maintaining standing culture conditions. CFU was determined and percentage survival was calculated based on CFU relative to the non-CuSO_4_ treated control group. The results showed that TbD1 intact WT and complemented *M. bovis* AF2122/97 ΔTbD1 (WT and ΔTbD1:TbD1) strains had significantly higher viability at 200 μM CuSO_4_ after 10-day incubation (Figure 4) than *M. bovis* AF2122/97 ΔTbD1. These results identified that the TbD1 locus of *M. bovis* AF2122/97 is important in protection against excess copper in a hypoxic environment.

### TbD1 Locus Is Involved in H_2_O_2_ Resistance in *Mycobacterium bovis* AF2122/97

**FIGURE 5:**
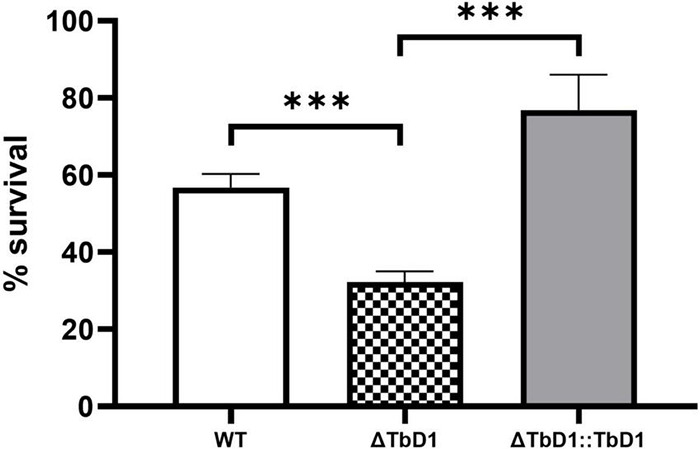
Oxidative stress experiments of *M. bovis* AF2122/97. H_2_O_2_ stress experiments with *M. bovis* AF2122/97 WT, ΔTbD1, and ΔTbD1:TbD1 strains to assess the survival ability of strains to 40 mM H_2_O_2_. Forty millimolars H_2_O_2_ was added into standing cultures for 1 h and then cultures were plated on 7H11 plates. CFU were then counted after 2–3 weeks of incubation and survival percentages were determined relative to the non-treated control. The data are shown as means ± SD and are representative of three independent experiments. Statistical significance of differences in survival percentages calculated using one-way ANOVA followed by the Dunnett’s *post hoc* test for multiple-comparisons (^***^p < 0.001).

Previous publications have presented conflicting conclusions in terms of oxidative stress tolerance of *M. tuberculosis* with an intact or deleted TbD1 locus. We therefore conducted oxidative stress challenges on our *M. bovis* AF2122/97 WT and TbD1 mutants, using rolling cultures to match methods used in these previous publications. Forty millimolars H_2_O_2_ was added to mid-log phase cultures of *M. bovis* AF2122/97 WT, ΔTbD1, and ΔTbD1:TbD1 strains and incubated for 1 h at 37°C, with percentage survival determined based on CFU relative to the control (no treatment). The results showed that *M. bovis* AF2122/97 with an intact TbD1 locus had significantly improved survival under H_2_O_2_ stress (Figure 5). Interestingly, we identified that *M. bovis* AF2122/97 had higher resistance to H_2_O_2_ stress than *M. tuberculosis* H37Rv. A previous study reported that *M. tuberculosis* H37Rv showed approximately 10% survival after 1 h exposure to a 10 mM H_2_O_2_ challenge (Bottai et al., 2020); our work confirmed this level of *M. tuberculosis* H37Rv survival to 10 mM H_2_O_2_, while we found that *M. bovis* AF2122/97 challenged with 10 mM H_2_O_2_ showed survival that was similar to the non-H_2_O_2_ treated control group (Supplementary Figure 1). Previously, Forrellad et al. (2019) found a higher resistance of *M. bovis* NCTC10772 to H_2_O_2_ relative to *M. tuberculosis* CDC1551, while Firmani and Riley (2002) reported that *M. bovis* Ravanel was more resistant than *M. tuberculosis* H37Rv to 5 mM H_2_O_2_. The gene encoding catalase-peroxidase, *katG*, has a SNP at position 463 that distinguishes *M. bovis* (463L) from *M. tuberculosis* H37Rv (463R) and other Lineage 4 strains (Heym et al., 1995; Stucki et al., 2016). Previous functional studies have, however, shown that this SNP does not lead to altered KatG activity (Saint-Joanis et al., 1999) excluding it as being important in H_2_O_2_ sensitivity. Hence, our data extends previous findings on H_2_O_2_ sensitivity, showing that *M. bovis* AF2122/97 is more resistant to H_2_O_2_ stress than *M. tuberculosis* H37Rv.

### RT-qPCR on BCG Denmark Further Validated RNA-seq Results and Highlighted the TbD1 Mediated Hypoxia-Specific Copper Pathway in *Mycobacterium bovis*

**FIGURE 6:**
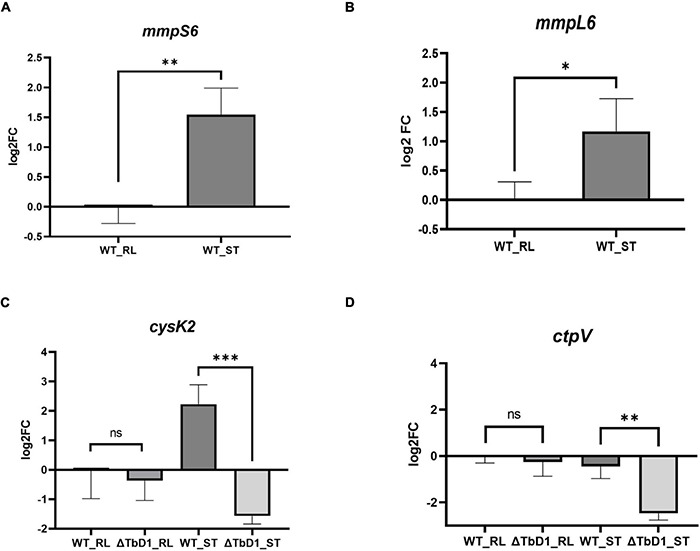
RT-qPCR validation on selected genes on BCG Denmark WT and ΔTbD1 under rolling and standing culture conditions. Log2 fold-change (log2FC) values were generated by comparing the expression of genes of BCG Denmark WT and ΔTbD1 strains under different culture conditions vs. WT under rolling condition control using 2 –ΔΔCt method, and expression levels of **(A)** *mmpS6*, **(B)** *mmpL6*, **(C)** *cysK2*, and **(D)** *ctpV* genes have shown. The data shown are means ± SD of gene expression from three independent biological replicates with duplicates, normalized with respect to 16S rRNA. Statistical significances of differences were calculated by Student’s *t*-test (*p < 0.05; ^**^p < 0.01; ^***^p < 0.001; ns, not significant) are shown.

Our RNA-seq data was obtained from *M. bovis* AF2122/97 WT and ΔTbD1 mutant strains. As a parallel way to validate the impact of the loss of TbD1 and the DEG findings, we constructed an independent mutant in the attenuated *M. bovis* strain BCG Denmark. RT-qPCR targeting several DEGs from the *M. bovis* AF2122/97 RNA-seq analysis (Supplementary Table 1) was performed on *M. bovis* BCG Denmark WT and ΔTbD1 strains. In agreement with our results from *M. bovis* AF2122/97, the expression of *mmpS6* and *mmpL6* in the BCG Denmark WT were approximately two to threefold higher in response to standing growth conditions than when grown rolling (Figures 6A,B). Our RT-qPCR results also showed induction of TbD1 expression by Triclosan induced oxidative stress in *M. bovis* AF2122/97 (Supplementary Figure 2), in agreement with a previous study in *M. tuberculosis* (Betts et al., 2003); hence our findings demonstrate that the TbD1 locus responds to changes in the redox status of *M. bovis*. The expression of two copper-responsive genes, *cysK2* (Figure 6C) and *ctpV* (Figure 6D) were also determined in BCG Denmark WT and ΔTbD1 in response to standing vs. rolling culture conditions. While there was no significant differential expression of *cysK2* and *ctpV* between BCG Denmark WT and ΔTbD1 strains when grown under rolling conditions, the two genes were significantly downregulated in BCG Denmark ΔTbD1 as compared to WT when grown using standing conditions. Thus, the RT-qPCR results on BCG Denmark WT and ΔTbD1 concurred with the *M. bovis* AF2122/97 RNA-seq data, revealing a link between the TbD1 locus in redox-state sensing and copper homeostasis.

### TbD1 Locus and Hypoxia-Specific Copper Sensitivity in BCG Denmark

**FIGURE 7:**
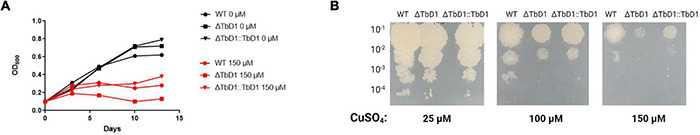
Contribution of TbD1 on BCG Denmark to Copper sensitivity. **(A)** BCG Denmark WT (circles), ΔTbD1 (squares), and ΔTbD1:TbD1 (triangles) strains were grown at 37°C in standing Sauton’s liquid media, and CuSO_4_ was added. Cultures were challenged with 0 μM CuSO_4_ (black symbols) or 150 μM CuSO_4_ (red symbols). Absorbance at 600 nm (OD_600_) was measured for 2 weeks. The data are representative of three independent experiments. **(B)** Drop assay determining the Copper resistance of TbD1 mutants. Serial dilutions (10-fold) of BCG Denmark WT, ΔTbD1, and ΔTbD1:TbD1 cultures were spotted onto 7H11 plates containing 25, 100, or 150 μM CuSO_4_. Data are representative of two independent experiments with replicates.

We next sought to use our BCG Denmark WT and ΔTbD1 strains to assess copper sensitivity and compare with our previous findings with *M. bovis* AF2122/97 WT and ΔTbD1. Analysis of BCG Denmark WT and ΔTbD1 strains in a basal Sauton’s liquid media supplemented with a range of copper concentrations revealed that the growth of BCG Denmark ΔTbD1 was inhibited by 150 μM CuSO_4_ (Figure 7A). Analysis of growth on solid media was assessed by drop assays on 7H11 agar plates containing 25, 100, and 150 μM CuSO_4_. These assays showed no apparent difference in growth of BCG Denmark WT, ΔTbD1, and ΔTbD1:TbD1 on plates with a low concentration of CuSO_4_ (25 μM) while BCG Denmark ΔTbD1 had limited growth at 150 μM CuSO_4_ with a smaller colony size. The BCG Denmark ΔTbD1 complemented with the TbD1 locus driven from its native promoter showed increased growth at 150 μM CuSO_4_ (Figure 7B).

### BCG Denmark ΔTbD1 Strains Expressed a Significantly Lower Level of Copper-Responsive Genes Under Copper Stress in Standing Condition and Contributed to Its Copper Sensitivity Phenotypes

**FIGURE 8:**
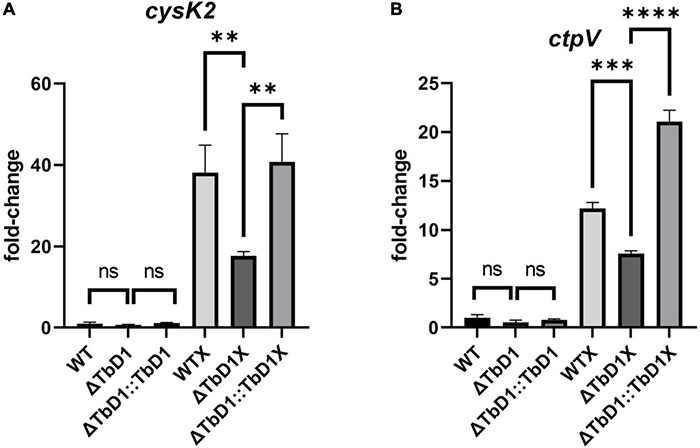
BCG Denmark ΔTbD1 copper response. BCG Denmark ΔTbD1 expressed copper-responsive genes at a significantly lower level under copper stress in standing culture conditions. Fold-change values were generated by comparing the expression of genes of WT, ΔTbD1, and ΔTbD1:TbD1 BCG Denmark strains treated with 100 μM CuSO4 for 3 h (marked as X) or untreated culture conditions vs. WT untreated control using 2 –ΔΔCt method, and expression levels of **(A)** *cysK2* gene and **(B)** *ctpV* gene have shown. The data shown are means ± SD of gene expression from three independent biological replicates with duplicates, normalized with respect to two reference genes *ftsZ* and *fbpB*. Statistical significances of differences were calculated by one-way ANOVA followed by the Dunnett’s *post hoc* test for multiple-comparisons (^**^p < 0.01; ^***^p < 0.001; ^****^p < 0.0001; ns, not significant) are shown.

To explore the sensitivity phenotypes that we observed in *M. bovis* AF2122/97 ΔTbD1 and BCG Denmark ΔTbD1 when grown under conditions of excess copper, we checked the expression level of several copper-responsive genes in standing BCG Denmark cultures after cultures had been incubated with 100 μM of CuSO_4_ for 3 h. To determine the candidate reference genes for this analysis the geNorm algorithm (Vandesompele et al., 2002) was used to examine the stability of eight potential reference genes identified from the literature (Supplementary Table 1). The geNorm analysis identified that optimal normalization would be obtained using the geometric mean of *ftsZ* and *fbpB*, and these were subsequently used in our gene expression analysis. We used cultures grown in basal Sauton’s media (without added CuSO_4_) as controls. Results showed that when cultured using standing conditions without copper, expression of *cysK2* and *ctpV* were not significantly different among BCG Denmark WT, ΔTbD1, and ΔTbD1:TbD1 strains. However, when cultured with a high concentration of copper (100 μM), the expression of *cysK2* and *ctpV* were significantly lower in BCG Denmark ΔTbD1 as compared to WT. Complementation of BCG Denmark ΔTbD1 with the WT locus (ΔTbD1:TbD1 strain) restored expression of *cysK2* and *ctpV* to WT levels (Figure 8). Hence, sensitivity phenotypes to copper stress that were observed in *M. bovis* ΔTbD1 strains were due to disturbed copper homeostasis caused by TbD1 knockout.

## Discussion

The function of TbD1 in *M. tuberculosis* strains has revealed conflicting results on the role of the locus in the oxidative stress response (Arumugam et al., 2019; Bottai et al., 2020). Hence while Arumugam et al. (2019) found that *M. tuberculosis* strains with an intact TbD1 locus had increased resistance to oxidative stress, Bottai et al. (2020) found that loss of the TbD1 locus conferred *M. tuberculosis* strains with increased resistance to oxidative stress. The reasons for these divergent findings are not immediately apparent, but may reflect differences in experimental protocols or strain genetic backgrounds. Our work did not seek to directly address these discrepancies, but rather to explore the function of TbD1 in *M. bovis* as a route to assess the locus’s functional role in animal-adapted tubercle bacilli. Hence, we used *M. bovis* WT and mutant strains as a basis for comparative transcriptomic studies. These studies indicated that in *M. bovis*, TbD1 mediated hypoxia-dependent copper response pathways.

Copper is an essential micronutrient, but it can be toxic at high concentrations as it catalyzes the formation of potent oxidants, hydroxyl radicals, from hydrogen peroxide *via* the Fenton reaction; these oxidants inhibit metabolic processes and damage DNA, lipids, and proteins (Kumar et al., 2011). A previous study described the transcriptional profile of *M. tuberculosis* after 3 h exposure to 500 μM CuCl_2_; 11 out of 30 copper-induced genes overlapped with genes identified in another study of cultures exposed to H_2_O_2_, showing a correlation between the oxidative and copper stress responses (Schnappinger et al., 2003; Ward et al., 2008). Copper can also interact with sulfur atoms in Fe–S cluster proteins and replace enzyme metal cofactors, thereby inactivating them at a high kinetic rate (Pope et al., 2013). *M. tuberculosis* is known to elaborate three independent pathways in response to excess copper. One is McdB which exports copper to the extracellular environment. The other two pathways are regulated by Cu^+^-responsive transcriptional repressors CsoR and RicR; RicR regulates genes controlled by six different promoters, while CsoR regulates a single four-gene operon (Darwin, 2015).

Our comparative transcriptomic study of *M. bovis* WT and ΔTbD1 mutant identified that multiple CsoR and RicR regulated genes were significantly downregulated in the ΔTbD1 mutant as compared to WT when strains were grown under standing culture conditions (that contain trace amounts of copper). These downregulated genes included *cysK2* which encodes an L-cysteine synthase that is involved in the biosynthesis of S-sulfocysteine, a precursor of mycothiol which acts as a redox buffer (Steiner et al., 2014). Other downregulated genes were *ctpV* which encodes a P1B-type ATPase that pumps copper cations to the cellular periplasm (Ward et al., 2010), and *mymT* that encodes a metallothionein that can bind up to seven copper ions with high avidity and hence increases copper tolerance (Rowland and Niederweis, 2012). Notably, we found that other enriched GO terms in our comparative analysis between mutant and WT included the *Cadmium ion response* GO term, a finding that might be explained by cross talk between copper ion response and cadmium ion response. Hence two genes in the *Cadmium ion response* GO term encoded CadI, which encodes a cadmium-induced protein, was found to be the highest induced gene after copper exposure (Hotter et al., 2001; Ward et al., 2008), and MymT which is a metallothionein that is induced by cadmium and copper (Gold et al., 2008). Interestingly, *cadI*, *cysK2*, and *ctpG* were also found to be induced by 0.5 mM of zinc in *M. tuberculosis* (Botella et al., 2011), showing the overlap between divalent metal ion responses in mycobacteria. Other GO terms that were revealed through DEG analysis related to Mo-molybdopterin cofactor metabolic/biosynthetic process (*moaA1*, *moaC1*, and *moaD1*) which are involved in the early steps of Mo-molybdopterin cofactor biosynthesis. Early steps in MoCo biosynthesis are linked to copper, iron, and metal homeostasis, as well as being linked with sulfur and cysteine metabolism (Williams et al., 2014). It is also possible that due to the downregulation of *cysK2*, accumulated thiosulfate stimulated the feedback expression of Moa1 locus *via* sulfur assimilation.

The effects of different culture conditions (standing vs. rolling) seen in our study highlights the importance of culture conditions when defining *in vitro* mycobacterial mutant phenotypes. Rolling is a common *in vitro* culture method for mycobacteria as it minimizes aerosol generation but maximizes aeration and yield. However, mycobacterial pathogens would not normally encounter such high aeration rates *in vivo*, but instead low oxygen tension is the more usual state during infection (Rustad et al., 2009). Hypoxia-based *in vitro* models are used as dormancy models and are thought to mirror some aspects of *in vivo* latent infection, with hypoxia being a key host-induced stress that limits the growth of tubercle bacilli and induces non-replicating persistence (Wayne and Sohaskey, 2001). Furthermore, in the context of reduced oxygen environments *in vivo*, it is known that hypoxia acts in concert with copper stress in the host macrophage (Via et al., 2008). The level of intraphagosomal copper concentration increases from 25 to 500 μM after phagocytosis of *M. tuberculosis*, but no such increase occurs after phagocytosis of less virulent mycobacterial species such as *M. avium* and *M. smegmatis* (Wagner et al., 2005). What’s more, previous studies have shown that copper exhibits greater toxicity under hypoxic environments because of the reduction of Cu^2+^ to more labile Cu^+^ under such conditions, and that the host macrophage increases copper uptake under hypoxia through upregulation of the macrophage copper importer Cu^+^ transport protein 1 (CTR1) (Beswick et al., 1976; White et al., 2009). Indeed, the effect is not specific to mycobacteria; Macomber and colleagues found that *E. coli* strains were more sensitive to copper under hypoxia (Macomber and Imlay, 2009). Also, previous studies proved that *Saccharomyces cerevisiae* and *E. coli* accumulate higher concentrations of copper in anaerobic cultures as compared with aerobic cultures (Strain and Culotta, 1996; Outten et al., 2001). Our findings corroborate the linkage of reduced oxygen to increased copper toxicity, with increased *in vitro* copper sensitivity of *M. bovis* ΔTbD1 when grown with standing culture conditions in liquid media and in drop assays on 7H11 plates. On this latter point of growth on solid media, it has been suggested (Beswick et al., 1976) that colonies on plates could experience reduced oxygen availability even with aerobically incubation, which may again potentiate the action of copper.

It is known that *M. tuberculosis* shifts to use lipids, including cholesterol, as a primary source of nutrition in the host (Bloch and Segal, 1956; McKinney et al., 2000; Pandey and Sassetti, 2008; Marrero et al., 2010). Furthermore, acidic pH and hypoxia are known to be a common stresses imposed on tubercle bacilli during infection (Wayne and Sohaskey, 2001). Notably, *in vitro* assays that seek to mimic *in vivo* stresses commonly encountered by *M. tuberculosis* have been shown to alter carbon metabolic flux, i.e., a recent study showed that lipids are the preferred primary carbon source in an acidic environment (Gouzy et al., 2021). GO analysis revealed an induction of genes involved in lipid metabolism in *M. bovis* AF2122/97 WT cultured under standing conditions. It’s thus perhaps not surprising that in our RNA-seq results, *in vitro* hypoxia was seen to trigger induction of *M. bovis* AF2122/97 genes involved in lipid-based metabolism; this likely shows that *M. bovis* senses hypoxia as a signal to adapt to the *in vivo* host environment. However, there were no significant DEGs for lipid metabolism between the WT and *M. bovis* ΔTbD1 mutant involved in either standing or rolling conditions. This observation suggests that the MmpS/L6 transport system is not involved in lipid transport *per se*. Indeed, the MmpL family has been shown to have diverse functions including uptake of nutrients, iron acquisition, or extrusion of toxins as stated. Furthermore, Bottai et al. (2020) used lipidomics assays on a set of WT and TbD1 mutants in an attempt to identify TbD1-associated lipids, but no clear difference between strains were observed.

It is also worth highlighting the AAC-AAG SNP in *mmpL6* which is only present in animal adapted strains. This SNP causes a non-synonymous N551K change located in the long α-helical hairpin on the PC subdomain of the protein (Supplementary Figure 1). Based on previous studies of RND transporters from Gram-negative bacteria, the PC subdomain is thought to contain binding sites for the exported ligands and have a role in substrate binding, hence determining substrate specificity (Elkins and Nikaido, 2002; Tikhonova et al., 2002). The presence of this SNP may have no functional significance, or may indicate subtle differences in substrate specificity of MmpL6 in animal-adapted lineages.

**FIGURE 9:**
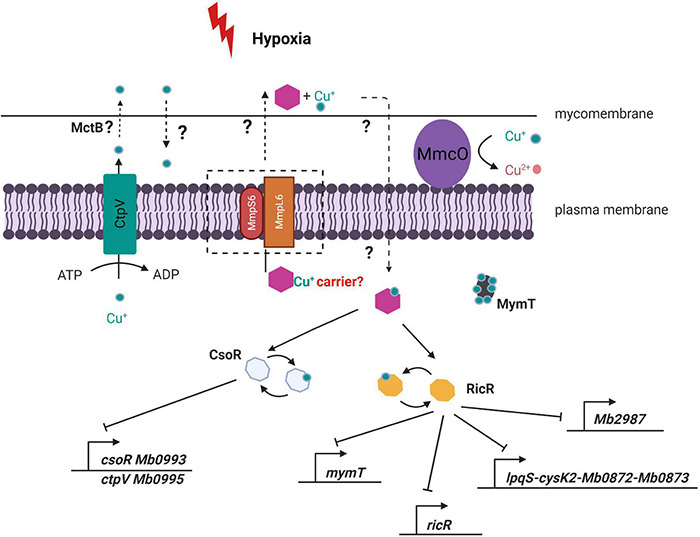
Model of potential consequences of ΔTbD1 disrupted copper homeostasis under hypoxia. The module suggests that the TbD1 locus mediates a hypoxia-specific copper response *via* translocation of an unidentified copper chaperone (purple hexagon) across the cytoplasmic membrane. The chaperone then transports copper ions into the cytoplasm with subsequent protein–protein interaction between the chaperone-copper complex and RicR/CsoR, allowing the expression of downstream proteins and promoting adaptive changes in intracellular copper homeostasis.

The mechanisms of copper acquisition and transport in mycobacteria are poorly identified, and copper chaperones have not been identified. As a way to conceptualize our findings, we hypothesize that the TbD1 locus may mediate a hypoxia-specific copper response by translocation of an (as yet unknown) copper chaperone across the cytoplasmic membrane. This potential system would have the chaperone transport copper ions into the cytoplasm with subsequent protein–protein interaction between the chaperone-copper complex and RicR/CsoR, allowing the expression of downstream proteins and promote adaptive changes in intracellular copper homeostasis (Figure 9). Notably *mmpl3* was found to be significantly downregulated under 0.5 mM zinc, while copper-responsive genes (i.e., *lpqS*, *cysK2*, and *mymT*) were induced in an *mmpl3* depleted *M. tuberculosis* mutant (Botella et al., 2011; Degiacomi et al., 2017). This indicates that other MmpL proteins are also be involved in metal responses, and suggests a possible fruitful area for future studies.

In conclusion, our findings provide insight into the function of the TbD1 locus in *M. bovis* and reveal a role in hypoxia-specific copper detoxification. Further experimentation will be required to elucidate whether the presence of an intact TbD1 locus favors *M. bovis* in adaptation to its preferred animal host.

## Data Availability Statement

The raw sequencing data are available in the Sequence Read Archive (SRA) under the BioProject ID PRJNA774648.

## Author Contributions

RM and SG designed the experiments and wrote the manuscript. RM performed the experimental work and data analyses. DF and GG contributed to bioinformatics analyses. JB contributed to RT-qPCR work. YS and CN contributed to experimental advice and interpretation. All authors approved the final submission.

## Conflict of Interest

The authors declare that the research was conducted in the absence of any commercial or financial relationships that could be construed as a potential conflict of interest.

## Publisher’s Note

All claims expressed in this article are solely those of the authors and do not necessarily represent those of their affiliated organizations, or those of the publisher, the editors and the reviewers. Any product that may be evaluated in this article, or claim that may be made by its manufacturer, is not guaranteed or endorsed by the publisher.
