# Corrigendum: The TbD1 Locus Mediates a Hypoxia-Induced Copper Response in *Mycobacterium bovis*

**DOI:** 10.3389/fmicb.2022.947450

**Published:** 2022-06-15

**Authors:** Ruoyao Ma, Damien Farrell, Gabriel Gonzalez, John A. Browne, Chie Nakajima, Yasuhiko Suzuki, Stephen V. Gordon

**Affiliations:** ^1^UCD School of Veterinary Medicine, University College Dublin, Dublin, Ireland; ^2^Hokkaido University International Institute for Zoonosis Control, Sapporo, Japan; ^3^UCD School of Agriculture and Food Science, University College Dublin, Dublin, Ireland; ^4^Division of Bioresources, Research Center for Zoonosis Control, Hokkaido University, Sapporo, Japan; ^5^UCD Conway Institute, University College Dublin, Dublin, Ireland

**Keywords:** tuberculosis, transcriptomic (RNA-seq), *Mycobacterium bovis*, phenotype, hypoxia, TbD1

In the original article, there was an error. It was stated that sodium pyruvate was added to *Mycobacterium bovis* AF2122/97 growth media to a final concentration of 10 mM, which is not correct; the correct concentration is 40 mM. A correction has been made to two sections of the **Materials and Methods**, **Bacterial Strains and Culture Conditions**, paragraph one:

“*Escherichia coli* strains that were used for plasmid propagation in MultiSite Gateway cloning (Life Technologies/Invitrogen/Thermo Fisher Scientific, Loughborough, United Kingdom) procedures were grown in LB medium or LB agar plates supplemented with selected antibiotics. Ampicillin (50 μg/ml), zeocin (25 μg/ml), and hygromycin (50 μg/ml) were added as required. BCG Denmark was grown in liquid Middlebrook 7H9 medium (Becton Dickinson, New Jersey, NJ, United States) supplemented with 0.05% Tween 80, 0.2% glycerol, 0.5% bovine serum albumin, 0.2% glucose, and 0.085% NaCl or 7H11 agar plates (Becton Dickinson, New Jersey, United States) supplemented with 0.2% glycerol, 0.5% BSA, 0.2% glucose, and 0.085% NaCl. *M. bovis* AF2122/97 was grown in the 7H9 and 7H11 media as described above, with 40 mM sodium pyruvate (Sigma-Aldrich, Ireland). When required, kanamycin, hygromycin, or zeocin were added to growth media to a final concentration of 50, 50, or 25 μg/ml, respectively. Standing cultures for RNA extraction were grown in 30 ml of 7H9 in 50 ml tubes (Sarstedt) with the caps tightly screwed, without shaking at 37°C. Rolling cultures for RNA extraction were grown in 30 ml of 7H9 in 850 cm^2^ roller bottles (Cellmaster), rolling at 2–3 rpm at 37°C. Sauton's medium was prepared using 4 g L-asparagine, 2 g citric acid, 0.5 g KH_2_PO_4_, 0.5 g MgSO_4_, 0.05 g ferric ammonium citrate, 0.1 ml of 0.01% ZnSO_4_, 60 ml glycerol, 2.5 ml of 20% Tween 80 in 900 ml deionized water and adjust pH to 7.0 with 1 M NaOH, adding 40 mM sodium pyruvate for *M. bovis* AF2122/97. The strains used in this study are listed in **Supplementary Table 1**.”

In addition, a correction has been made to the **Materials and Methods**, **Drop Assays and Copper Challenge**, paragraph one:

“For drop assays, BCG Denmark WT and TbD1 mutants were scaled up to OD_600_ = 0.8–1.0 as described above. Cultures were then pelleted by centrifugation and washed twice with Sauton's media and resuspended in Sauton's media to OD_600_ = 0.1. Ten-fold serial dilutions were conducted with each culture on a 96-well plate. Six-microliters drops were spotted onto 7H11 agar plates with increasing concentrations (25, 100, and 150 μM) of CuSO_4_ in replicates and incubated at 37°C for 14–16 days. For copper challenge in liquid media, cultures were collected, washed, and resuspended as described above and 150 μM CuSO_4_ was added. The OD_600_ were then read constantly over 14 days. To check the cell viability after copper stress was imposed on *M. bovis* AF2122/97, the strains were grown in 7H9 with 40 mM sodium pyruvate to OD_600_ = ~0.8 and cultures were then pelleted by centrifugation, washed twice with Sauton's media and resuspended in Sauton's media to OD_600_ = 0.1. Cultures then were maintained in the absence (control) or presence of 200 μM CuSO_4_ in standing conditions for 10 days. Cultures were plated out at day 10 on 7H11 plates. CFU were determined after 2–3 weeks incubation. The viability was expressed as a percentage of survival, calculated as the ratio between the CFU recovered from cultures exposed to 200 μM CuSO_4_ over those obtained from unexposed cultures.”

The authors apologize for this error and state that this does not change the scientific conclusions of the article in any way. The original article has been updated.

## Publisher's Note

All claims expressed in this article are solely those of the authors and do not necessarily represent those of their affiliated organizations, or those of the publisher, the editors and the reviewers. Any product that may be evaluated in this article, or claim that may be made by its manufacturer, is not guaranteed or endorsed by the publisher.

