# Supplementary material for: The TbD1 Locus Mediates a Hypoxia-Induced Copper Response in Mycobacterium bovis
Source: Front Microbiol. 2022 Apr 14;13:817952. doi: 10.3389/fmicb.2022.817952 (PMC9048740; doi:10.3389/fmicb.2022.817952)
Supplement: Supplementary file 8 [file Data_Sheet_1.pdf]

The TbD1 locus mediates a hypoxia-induced copper response in *Mycobacterium bovis*

Ruoyao Ma, Damien Farrell, Gabriel Gonzalez, John A. Browne, Chie Nakajima, Yasuhiko Suzuki, and Stephen V. Gordon

Supplementary Figures

Supplementary Figures

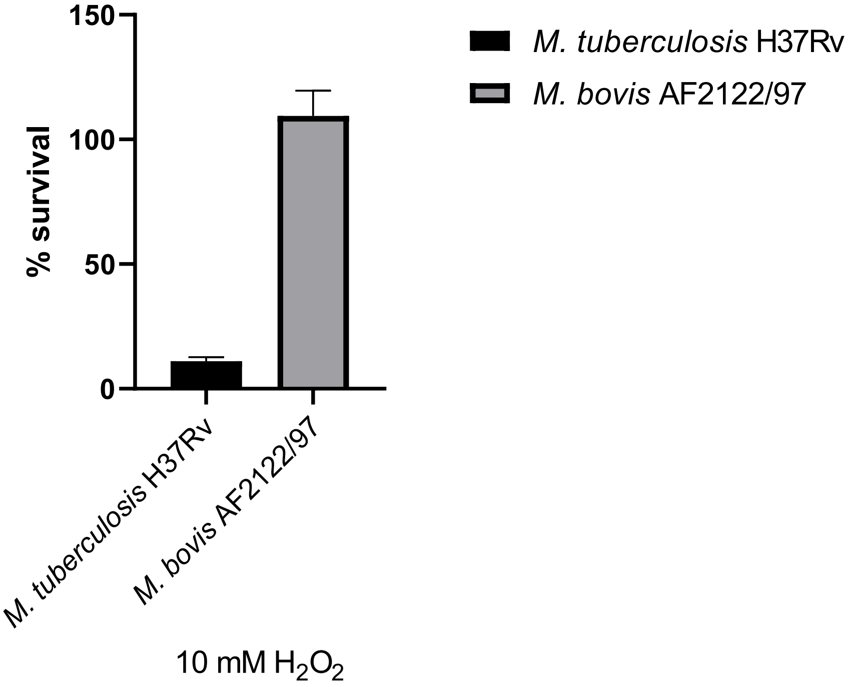

Supplementary Figure 1. 10 mM H_2O_2 challenge on *M. tuberculosis* H37Rv and *M. bovis* AF2122/97. H_2O_2 stress on *M. tuberculosis* H37Rv and *M. bovis* AF2122/97 strains to assess the survival ability of strains to 10 mM H_2O_2. 10 mM H_2O_2 was added into standing cultures for 1h and then cultures were plated on 7H11 plates. CFU were then counted after 2-3 weeks of incubation and survival percentages were determined relative to the non-treated control. The data are shown as means ±SD and are representative of three independent experiments.

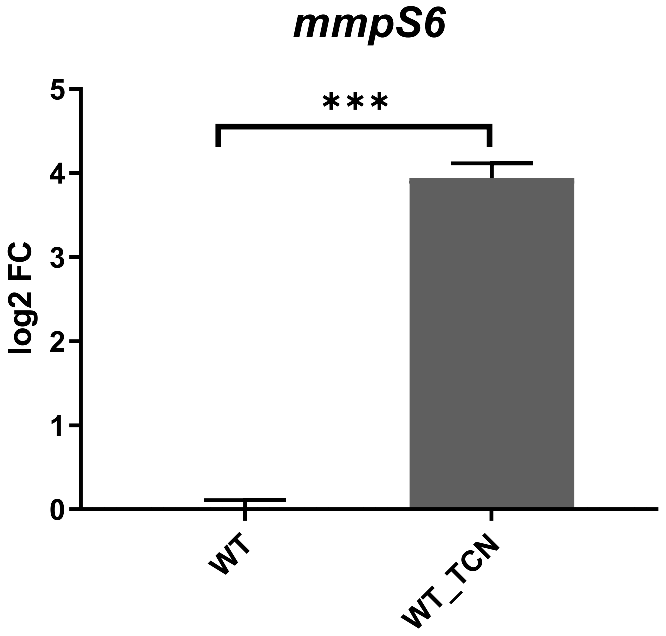

Supplementary Figure 2. Oxidative stress induced the expression of *mmpS6* in *M. bovis* AF2122/97. Log2 fold-change (log2FC) values were generated by comparing the expression of genes of *M. bovis* AF2122/97 WT strains with Triclosan treatment (16 ug/ml TCN for 3h) vs. non-treated control using 2 –ΔΔCt method. The data shown are means ±SD of gene expression from three independent biological replicates with duplicates, normalized with respect to 16SrRNA. Statistical significances of differences were calculated by Student's t test (*p <0.05; **p <0.01; ***p < 0.001; ns: not significant) are shown.

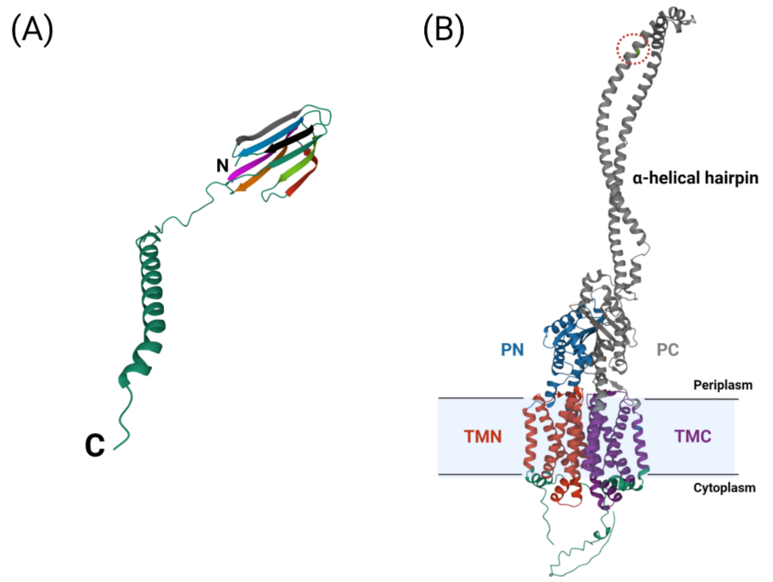

Supplementary Figure 3. Overall 3D protein structure of MmpS6 and MmpL6 from *M. bovis.* The 3D structure prediction of MmpS6 and MmpL6 proteins were done using a computational method, AlphaFold, which uses a neural network-based model and trained by evolutionary, physical and geometric constraints of protein structure (Jumper et al., 2021)**. (A)** Cartoon depiction of the MmpS6 crystal structure. Tansmembrane α-helix is coloured green and β-strands are coloured with different colours. **(B)** Cartoon depiction of the MmpL6 crystal structure. Tansmembrane N-terminal domain (TMN) is coloured red, tansmembrane C-terminal domain (TMC) is coloured purple, porter N-terminal domain is coloured blue and porter C-terminal domain is coloured grey with the long α-helical hairpin is indicated. The 551 aa Asparagine (N)- Lysine (K) location is also marked out in green which is inside of the red dotted circle.

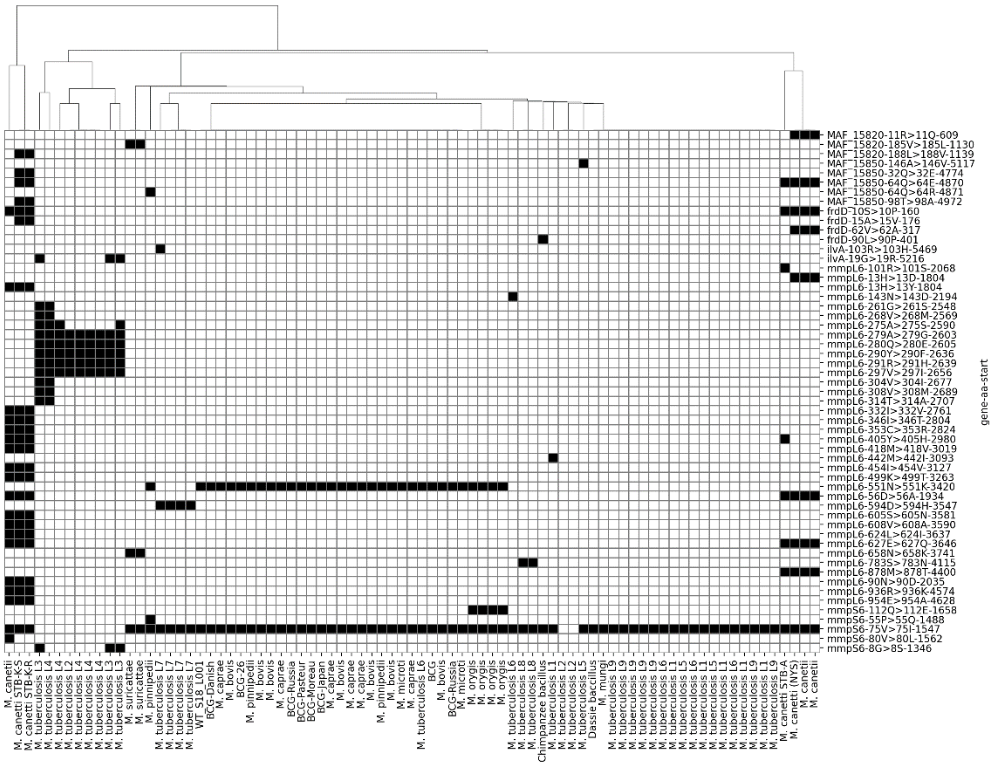

Supplementary Figure 4. SNP calling in TbD1 locus across MTBC. The clustered heat map constructed from SNP calling results by SNiPgenie on 81 samples representing different lineages across MTBC (Table S7). The columns represented the samples and the rows corresponded to SNP location and effects.

References

Jumper, J., Evans, R., Pritzel, A., Green, T., Figurnov, M., Olaf, R., et al. (2021). Highly accurate protein structure prediction. doi:10.1038/s41586-021-03819-2.
